# Central precocious puberty and psychiatric disorders: a call for deeper inquiry

**DOI:** 10.1097/MS9.0000000000003878

**Published:** 2025-09-15

**Authors:** Zahabia Adnan, Hooriya Mahmood, Hermann Yokolo

**Affiliations:** a Department of Medicine, Jinnah Sindh Medical University, Karachi, Pakistan; b Department of Research, Medical Research Circle (MedReC), Goma, DR Congo

*Dear Editor,*

We have reviewed the article “Central precocious puberty and psychiatric disorders”[1], which explores the association between early pubertal onset and increased vulnerability to psychiatric disorders. However, we would like to address additional considerations that can further strengthen the study’s reliability and credibility. This article complies with the TITAN 2025 guidelines – repressing the reporting and use of AI[2].

First, the article overlooks how early-onset puberty affects brain development in children with central precocious puberty (CPP). Early hormonal exposure can cause the brain to mature faster than the child’s actual age, making them vulnerable to emotional instability. Longitudinal neuroimaging assessments are lacking and are important to understand the influence of early hormonal shifts on brain development. Spielberg *et al* discovered that higher testosterone levels in teens weaken orbitofrontal cortex (OFC)–amygdala links, which increase emotional reactivity[3].

Additionally, there is a lack of discussion on GnRH agonist (GnRHa) treatment, which is standard for CPP. Although a strong link between CPP and its psychiatric outcomes is shown, it overlooks how medically delaying puberty can impact socioemotional development. A cross-sectional fMRI study revealed that GnRHa-treated girls with CPP showed stronger emotional responses to fearful faces than their peers[4].

Furthermore, the study acknowledges a unidirectional relationship between CPP and psychiatric disorders, yet it fails to consider potential bidirectional pathways. It does not address the fact that early-life stress can induce early onset of puberty by disturbing the regular activity of the Hypothalamic–Pituitary–Adrenal (HPA) axis. For example, prenatal stress has been associated with elevated cortisol levels in children at 4.5 years, which causes earlier menstruation and poorer psychological well-being[5]. The intricate developmental interactions are oversimplified by neglecting these factors.

Lastly, prevalent neurodevelopmental conditions like Attention Deficit Hyperactivity Disorder (ADHD) and autism are not considered, which are often associated with behavioral issues. By excluding these factors, the article may overstate the psychiatric issues to CPP alone, missing other contributors. A Korean study reported that children with ADHD are twice as likely to develop CPP, likely as a result of similar neurological changes caused by the stress and HPA axis dysregulation[6].

In conclusion, addressing these gaps would lead to the study’s viability to a more advanced level. A more comprehensive exploration of early hormonal changes, treatments like GnRH agonists, discussing bi-directional pathways of this relationship, and incorporation of longitudinal studies for better results of CPP would provide a more balanced perspective about the relationship between CPP and psychiatric disorders. Addressing these limitations can provide an effective approach to treating psychiatric conditions related to CPP.

## Data Availability

Not applicable.
